# Knowledge About Bullying by Young Adults With Special Educational Needs With or Without Disabilities (SEN/D)

**DOI:** 10.3389/fpsyg.2020.622517

**Published:** 2021-01-08

**Authors:** Víctor González-Calatayud, Marimar Roman-García, Paz Prendes-Espinosa

**Affiliations:** ^1^Department of Statistics, Mathematics and Computer Science, Miguel Hernández University of Elche, Elche, Spain; ^2^Department of Didactic and School Organization, University of Murcia, Murcia, Spain

**Keywords:** bullying, cyberbullying, disability, special education needs, professional development, teachers’ training

## Abstract

Bullying of people with Special Educational Needs with or without disabilities (SEN/D) is a reality, being one of the most affected groups. This study presents the data obtained in a European Erasmus+ project in which 96 young people and adults with SEN/D from four countries participated: Ireland, Spain, Italy, and Portugal. Firstly, a questionnaire was passed to see the general knowledge of these people in relation to bullying. Then a training program was carried out and finally the questionnaire was passed again to see if the data improved. From the data it appears that people with SEN/D lack skills, knowledge, and resources to deal with bullying. After the completion of the training program the data obtained from the questionnaire improved in many respects. Sometimes people with SEN/D are not aware that they are suffering from this type of situation due to lack of knowledge, so it is necessary to continue implementing training programs to help improve this situation.

## Introduction: Definition of Disablist Bullying

Since Olweus (1979) introduced school violence as a field of study, multiple labels have been used to name this problem according to the different countries in which it has been studied along with their different cultural and linguistic conceptions. In the last 30 years it can be seen how the international scientific community adopts the English word, “bullying” as the most appropriate term to refer to school violence between peers (Ortega, 2010). The same consensus currently exists to define this problem, understanding as bullying in those situations in which at least the following three characteristics are present: intentionality, duration-repetition, and power imbalance between victim and bully (Olweus, 1999; Erdur-Baker, 2010; Menesini and Salmivali, 2017).

However, the focus of this study is on a specific group of victims of bullying. Specifically, we focused on the bullying received by people with special educational needs (SEN). This type of bullying is commonly known as “disablist bullying.” Disablist bullying can be defined as the harassment that people with special educational needs with or without disabilities (SEN/D) receive because of their own condition. It can be carried out by people with or without disabilities and includes other types of harassment such as physical, verbal, social, psychological, or cyber-bullying. As in cases of traditional bullying, there must be repetition, intentionality, and an imbalance of power between the victim and the bully(s).

This study is part of an Erasmus+ project funded by the European Union called “Disabuse – disablist bullying: experience into change, providing the right support service.” Professionals from Ireland, Italy, Portugal, and Spain have participated in this project with the aim of offering people with SEN and the professionals who work with them resources for the prevention and solution of bullying.

### Current Situation Regarding the Bullying of People With SEN

The first reference we found mentioning bullying of children with SEN, known today as disablist bullying, was published in 1989. O’Moore and Hillery (1989) highlighted for the first time the increased exposure to bullying behavior of children with SEN and referred that the frequency is twice as high as for the non-SEN children. Five years later, Thompson et al. (1994) published a paper reporting the incidence of bullying behaviors among children with special needs in mainstream schools. They concluded that those students with made statement were more likely to be a victim of bullying. These results have been confirmed since then, with other researchers and studies in the last 30 years.

Blake et al. (2012) analyzed the prevalence of bullying victims among students with disabilities in the United States and this study established that 24.5% students in elementary school and 34.1% in high school were bullied. This is one to one and a half times the national average for students without disabilities. In addition, this study found that those people with disabilities who had been bullied once, were at greater risk of being bullied again. In general, students with autism at both levels and high school students with orthopedic impairments were more at risk of repeated victimization experiences. Menesini and Salmivali (2017) analyzed data from over the world and they found that students with more risk to bullying are: people with disabilities, suffering from obesity, or the ones belonging to ethnic or sexual minorities.

Some studies have detected that students with autism spectrum disorder (ASD) are the ones who suffer most from bullying because of their status as people with SEN. Schrooten et al. (2017) revealed in their study that these people were the most vulnerable to being in bullying situations compared to their peers without ASD. In the same line, the Spanish Autism Confederation in 2016 has already declared that students with ASD are more at risk of being bullied. Specifically, almost half of the students with ASD experience bullying, so they are four times more at risk of being bullied as compared to the general population.

The meta-analysis carried out by Pinquart (2017) concludes that children and adolescents with chronic physical illness or disability were more likely to be victims of bullying in general [odds ratio (*OR*) = 1.65], particularly physical bullying (*OR* = 1.47), relational bullying (*OR* = 1.47), verbal bullying (*OR* = 1.67), cyberbullying (*OR* = 1.39), and illness-specific teasing (*OR* = 5.29).

In the finding presented by González-Contreras (2017), with 1077 students between the ages of 11 and 13, he concluded that victims with SEN face harassment more frequently than students without SEN (22.7% vs. 17.8%). Naylor et al. (2012) prepared a table identifying the risk of bullying according to the type of SEN, with people with SEN being the most at risk of being bullied (Table 1). Bourke and Burgman (2010) investigated 10 cases of study (with children aged 8–10 years) in Australian schools and they highlighted the fact that children with disabilities suffer all types of bullying (physical or psychological) and remarked about the positive effect of the support from all friends, parents, and teachers.

**TABLE 1:** Risk of abuse in students with different disabilities (Naylor, Dawson, Emerson, Tantam, and Walters).

**SEN/D**	**Risk**
Autism Spectrum Disorder (ASD)	More than 7.0 times
Speech, language, and communication problems	More than 5.3 times
Multiple disabilities	More than 3.3 times
Behavioral, emotional, or social difficulties	More than 3.3 times
General learning disorder	More than 2.6 times
Physical disability	More than 2.4 times
Specific learning disabilities	More than 1.4 times
Sensory deficit	More than 0.5 times

Maguire et al. (2019) considered that students with intellectual disabilities are one of the most vulnerable sections of students in ordinary schools and even in special schools. They developed and piloted a training program in secondary schools and demonstrated that teachers’ training is probably one of the most important factors to reduce bullying in schools because they may have greater influence, and thus, be able to promote positive attitudes toward SEN students in other pupils.

In the case of adults, Jenaro et al. (2018) investigated cyberbullying among adults with intellectual disabilities, aged 18–40 years. They concluded that 15.2% of the participants have been cyberbullied. The main reason the authors outlined was “being different,” with verbal aggression being the most common cyberbullying behavior. They proposed more research and the implementation of prevention programs. Moreover, Christensen et al. (2012) confirmed that adolescents with intellectual disability are at the risk of being bullied more than their peers, especially if they have lower social skills.

Similarly, Bear et al. (2015) used a large sample of 1026 parents of children with disabilities and 11,500 parents of children without disabilities. These authors stated that rates differ in the function of two factors: the disability type and criteria used to classify students as being bullied or not. Regarding the first criteria, the authors concluded that “students with disabilities are generally at greater risk of being bullied compared with children without disabilities,” but these differences are clearer for children with emotional disturbance, health impairments (including attention deficit hyperactivity) and ASD. They explained these results because behavior problems and social skill deficits are more frequent in these cases. On the other hand, the second problem is the criteria used to define bullying and the measures used by researchers. Between bullied and non-bullied, there is a wide gap with lot of different cases.

However, Fink et al. (2015) did not find a higher probability of facing harassment in people with SEN compared to their peers without it; although when controlling for the variables of extant behavioral and emotional difficulties, the likelihood of reporting victimization increased. The same is true on the work of Martos and del Rey (2013) who, after observing harassment in a sample of 627 high school students, ruled out the existence of significant differences between students with and without SEN.

### Experiences and Projects About Disablist Bullying

There is a large body of research addressing the study of the prevalence of bullying and cyber-bullying at school; however, there are only a few studies that focus on analyzing the problem when the victim of the bullying is a person with SEN; there are even less knowledge and attitudes that people with SEN have about bullying and cyber-bullying situations. There are a lot more initiatives and associations that are concerned about the bullying associated with SEN or other topics like gender, or race. For instance, Mind^1^ or Show Racism the Red Card^2^ both in United Kingdom; SeeMe^3^ in Scotland; or AEPAE^4^ in Spain. It is relevant to consider some projects and real experiences which focus on the detection and prevention of bullying in the field of diversity, for instance:

• The “European Anti-bullying Network^5^” funded under the Daphne Program III of the European Commission. It is a work group with 19 members from 15 European countries engaged with the prevention and tackling of bullying. They have a digital library of resources and different activities for students, teachers, and parents.
• The project “I Am Not Scared”^6^ funded by the European Union Lifelong Learning Program involved 11 partners in nine different European countries. The aim is to involve educational agents (teachers, students, parents, counselors, and policy makers) in the prevention of bullying and promote a European strategy to prevent and tackle bullying.
• The Erasmus+ project “Sonet-Bull”^7^ promotes the use of social networking ICT tools to deal with student bullying and they also work with teachers, parents, and stakeholders under the idea of collaboration to deal with bullying at schools. They also have a digital repository and an online training course.
• “Eubully” project^8^ has been funded by Daphne Program of the European Union and they have designed some apps for use on mobile technologies to support teachers and school staff to be proactive. It involves SEN students as part of marginalized groups in a blended approach based on drama strategies.
• “incluD-ed – European Network on Inclusive Education^9^” is a network co-financed by the European Social Fund around good practices for on inclusive education for people with disabilities. This project has a digital repository with some practices connected to bullying.

The following are the results obtained after the experience carried out in the framework of the Erasmus+ Project “Disabuse Disablist Bullying- Experience into Change, providing the right support services” (2017–2019) financed by the European Union.

### DisAbuse Training Program

The training program was designed by the research team in Ireland and was subsequently reviewed by the other members of the project. The objectives of the training program were:

• Understand what bullying is in all its forms and become aware of the differences between conflict and bullying.
• Understand what cyber-bullying is and the many different ways that it can happen.
• Understand what relationship bullying is and how it can manifest within environments that are restrictive.
• Understand the relationship between bullies, victims, and bystanders and come to realize that bullying is a group issue.
• Understand what to do and how to deal with bullying behaviors directed toward themselves and how to offer support to others.
• Learn about the importance of growing your own self-esteem and the importance of self-advocacy.
• Learn about problem-solving and the many ways that we can help ourselves to work out our daily living relationship difficulties.
• Learn about people’s right to making a complaint and how it needs to work.

On the basis of these objectives, a manual was drawn up for the trainers of people with SEN/D in which the following blocks were worked on: (1) What is bullying?, dealing with cyberbullying; (2) understanding bystanders, bullies, and victims; (3) what is empathy and respect; (4) what to do about bullying; and (5) staying strong against bullying.

The methodology used is based on putting the participants at the center of the teaching-learning process. Therefore, based on a brief explanation, the work was focused on carrying out different dynamics depending on the content so that the participants worked in different ways: through debate, case analysis, role playing, etc. The courses were held between March and April 2019. At least one session was held for each of the blocks, with this point being flexible depending on the context of each country and the needs of participants. The training manual can be found on the project page^10^.

## Materials and Methods

During the development of this project, a training activity was carried out in relation to harassment addressed to people with SEN, specifically a course in which people with SEN from different European countries (Ireland, Italy, Spain, and Portugal) could learn to know this problem better and acquire skills to prevent it and also to act in situations of harassment. We have assessed the experience with a pre-test and post-test research design from a quantitative approach. This paper presents the information gathered previously (pre-test) about the knowledge, attitudes, and opinions that the participants in the course had about bullying and cyberbullying; and also the information collected after the training period (post-test) with the same topics of interest. We have used a questionnaire *ad hoc* and we made some adaptations of the questionnaire in relation to the different disabilities of the studied population.

### Objectives

This study wanted to help tackle disablist bullying by learning from the experiences of both young people and adults with SEN/D. Based on research and good practices, a course and some specific educational resources were designed to help to empower these young people and adults to take charge around the issue of disablist bullying and to stop it from happening. The objectives of this study were to analyze, firstly, the knowledge about school bullying and the skills to prevent or solve this problem, and secondly, to observe whether the training program described above improved that knowledge and those skills of the participants.

### Participants

In the initial round a total of 96 questionnaires were collected from participants located in the four countries. Of these, 52 were girls and 44, boys. Twenty-one respondents were from Ireland, 17 from Italy, 19 from Portugal, and 39 from Spain. Of the participants, 63.5% were 23 years or older, 12.5% between 21 and 22 years, 15.6% between 18 and 20 years, and 8.4% between 15 and 17 years. In terms of the SEN indicated by the participants it was noted that: 63.54% had intellectual disabilities of various kinds, 10.42% sensory disabilities, 8.33% various learning disabilities, 3.13% motor disabilities, 3.13% multiple disabilities, 2.08% ADHD and 2.08% ASD. A total of 7.29% did not indicate which disability they had. In terms of the type of center they attend, it was found that: the majority were in support services (32), followed by public school (13), and work (7), with “Others” chosen by 35. More than half of the participants indicated that their disability is intellectual.

After the training, 78 questionnaires were collected, so 18 participants did not complete the post-test after the end of the training program.

### Instruments

The creation process has been rigorous according to McMillan and Schumacher (2001). In the first place, the following five blocks that were included in the initial project proposal, were considered: (1) attitudes and personal opinion; (2) personal experience; (3) skills to prevent and solve; (4) knowledge about bullying; and (5) levels of direct interaction/resources/organization. From these blocks, we made a first draft of the possible questions. These questions were developed from the most current version of the manual created for the project. These initial questions were sent to the different participating teams to critique using expert judging technique based on the Delphi method. In this phase, we received eight answers from individual experts. The participants in this phase were experts on bullying, SEN, cyber-bullying, and education.

Once all the suggested changes had been made, a group discussion was held during one meeting to improve the questionnaires and reduce the number of items. In this second phase of validation, 10 people participated, and we used group dynamics to organize the discussion group in a sequence with three steps: individually, in small group work (two or three people) and in big group work to reach a final agreement. In the end, a questionnaire was obtained with a total of 31 dichotomous items (yes or no) addressed to students and adults with SEN. The questions were grouped into the five blocks indicated above. The items included certain images that could help understand the issues. Cronbach’s alpha coefficient was 0.853 which confirms the reliability of our instrument.

### Procedure

Before the realization of the course, the project partners created a training manual that included dynamics that allow people with SEN to be trained in the prevention and treatment of situations of bullying that may arise. Institutions and associations in charge of training and assisting people with SEN were contacted. Those who wanted to participate in the course were asked to complete a questionnaire before. During the process of answering, the evaluators assisted the participants to help them answer it. After 3 months with different sessions of training in every country, we used the questionnaire to collect data of post-test in the same way as in the pre-test, using printed questionnaires and being helped by the monitors. This research had all the ethical requirements, and all the permissions were signed by the participants.

### Data Analysis

The statistical program SPSS 22.0 was used to perform the analyses. Descriptive analysis was performed where the frequencies in the items and the mean in the global factors were analyzed. Before the differential analysis, the homogeneity and distribution of the sample was analyzed, verifying that these criteria were met. The Students *t*-test was carried out for independent samples to analyze the difference between items and ANOVAs to know the difference between age groups. The significance level of 0.05 was used. Cohen’s *D* (0.02 low effect, 0.05 medium effect, 0.08 large effect) and eta square (0.01 low effect, 0.06 medium effect, 0.14 large effect) were used to analyze the effect size.

## Results

In this part of the article we will explain the most relevant data obtained from pre-test and post-test. After, we reflect about the differences to explain the impact and the results of the training experience.

### Attitudes, Personal Opinion

The first category that was evaluated refers to the attitudes that students with SENs have toward bullying and cyberbullying and what their personal opinion is (Table 2).

**TABLE 2:** Results on participants’ attitudes and opinions about bullying.

	**Yes**	**No**
I think that bullying is a normal thing that happens to people	42.7%	57.3%
I try to avoid or ignore bullying when I see it	59.4%	40.6%
People with SEN/D are more at risk of bullying than other people	77.1%	22.9%
When people use the Internet, they are more at risk of being bullied	88.5%	11.5%
A bully does not care about how they are making their victims feel	64.6%	35.4%
It is ok to take advantage of people who are not as powerful as you or as able as you	18.8%	81.3%

Overall, 42.7% of participants considered bullying to be normal. Whereas, more than half (59.4%) indicated that they tried to avoid or ignore bullying when they saw it. Most believe that people with SEN/D were at greater risk of being bullied, just as Internet use increases the risk of being bullied. Moreover, slightly more than 60% felt that the bully does not care about the victim’s feelings.

Considering the overall factor, the data show a high average of 4.25 (*SD* = 1.24). No gender differences were found in this variable, *t*(90.98) = 0.259, *p* = 0.797, *d* = 0.01, although the mean was slightly higher for women (4.26, *SD* = 1.21). As for age, no significant differences were found, *F*(1,3) = 1.23, *p* = 0.30, μ^2^ = 0.02, even though the mean was higher for the age group 15–17 years (*M* = 4.31, *SD* = 0.91) and older than 23 (*M* = 4.32, *SD* = 1.22), and lower between 21 and 22 years (*M* = 4.04, *SD* = 1.24).

### Personal Experience

As shown in Table 3 above, a high percentage of participants (68.8%) claimed to know people who have been harassed/bullied. Moreover, only 34.4% had taken courses on bullying, so they probably need more training to prevent these intimidating situations. A total of 44.8% said that it is not their problem when people bother or ignore someone.

**TABLE 3:** Results on the personal experience of the participants in relation to bullying.

	**Yes**	**No**
I know people who have been bullied	68.8%	31.3%
I have done courses about bullying	34.4%	65.6%
It is none of my business when people get picked on or left out of a group	44.8%	55.2%

In the global factor, the score presents average data, *M* = 2.26, *SD* = 0.781. No differences were found between men and women, *t*(94) = 0.239, *p* = 0.811, *d* = 0.02, being the average between men (*M* = 2.25, *SD* = 0.876) and women (*M* = 2.27, *SD* = 0.699) very similar. In relation to age, no significant differences were found, *F*(1,3) = 1.201, *p* = 0.314, μ^2^ = 0.03. Although the differences are not significant, the means differ between age groups. The mean was higher in the 15–17 age range, being 2.44 (*SD* = 0.835), followed by over 23 years (*M* = 2.28, *SD* = 0.786), 18–20 years (*M* = 2.2, *SD* = 0.635), and 21–22 years (*M* = 2.12, *SD* = 0.866).

### Skills to Prevent and Cope

This block asked essential questions about the prevention of bullying (Table 4). As can be seen in the table above, 89.6% consider that they get along with everyone, which is a protective factor. The same applies to observers of these situations, a key role in maintaining the situation, as 92.7% indicate that if they saw this type of situation, they would comment on it to the person in charge. However, 25% do not know how to say no when a colleague or another person asks them to do something they do not want to do.

**TABLE 4:** Participants’ prevention and cope skills.

	**Yes**	**No**
I get along with pretty much all my classmates/people in my life	89.6%	10.4%
The bullying rules that we have in school/college/training center/day center are important	95.8%	4.2%
If a classmate or friend makes an annoying comment to me or makes me feel upset, I’ll tell them	83.3%	16.7%
If I see a classmate or friend being annoying or disrespectful, I will tell someone in charge or someone I trust	92.7%	7.3%
All my classmates and friends respect me for who I am	86.5%	13.5%
I should respect all of my classmates/people in my life	97.9%	2.1%
When I have problems with my friends, we can solve it without fighting	92.7%	7.3%
I know how to say no when a classmate or someone asks me to do something and I don’t want to do it	75%	25%

Within the global factor, the average obtained was 5.06 (*SD* = 1.09). Statistically significant differences were found in relation to sex, *t* (59.97) = −1.99, *p* = 0.05, *d* = 0.25, being in this case the mean of females lower (*M* = 4.94, *SD* = 0.68) than males (*M* = 5.21, *SD* = 1.4). As for age, no significant differences were obtained, *F*(1,3) = 0.045, *p* = 0.987, μ^2^ = 0.02.

### Knowledge About Bullying

In the fifth block (Table 5), the participants were asked about specific aspects he was going to work on during the course. The table shows that 62.5% know the difference between harassment and conflict. Regarding the essential elements of bullying, 84.4% know that there is an imbalance of power, 83.3% understand that there must be intentionality and 89.6% know that repetition is necessary. However, 36.1% believe that when someone around them bullies them, it is bullying.

**TABLE 5:** Results in relation to knowledge about bullying.

	**Yes**	**No**
I know the differences between having a conflict with someone and being bullied	62.5%	37.5%
Bullying only happens to children	15.6%	84.4%
Bullying only happens in schools	17.7%	82.3%
Bullies may have behavior problems and low self-esteem	82.3%	17.7%
Bullying is a power imbalance, the bully wants to be in charge	84.4%	15.6%
Bullying is when someone wants to hurt you on purpose by doing or saying something	83.3%	16.7%
Bullying is when your boss, teacher, good friend, support staff, or parents tell you off because you have done something that you should not have done	36.1%	63.5%
Bullying is when someone is singled out and picked on time and time again	89.6%	10.4%
Disablist bullying can be also called discrimination	85.4%	14.6%

As for the knowledge factor, the average is 6.21 (*SD* = 1.4). In this factor no differences were found in terms of sex, *t*(94) = −0.76, *p* = 0.45, *d* = 0.08, with the mean for females (*M* = 6.16, *SD* = 1.22) and males (*M* = 6.27, *SD* = 1.59) being very similar. There were also no differences in terms of age, *F*(1,3) = 1.68, *p* = 0.18, μ^2^ = 0.01 although the 21–22-year-old range shows a considerably lower average (Table 6).

**TABLE 6:** Age-related differences in the “knowledge about bullying” dimension.

	***N***	**Mean**	**SD**
Between 15 and 17	8	6.12	1.03
18–20	15	6.16	0.82
21–22	12	5.83	1.15
+23	61	6.31	1.55
Total	96	6.21	1.4

### Levels of Direct Interaction/Resources/Organization

As shown in the table above (Table 7), only 62.5% know what to do if a bullying situation occurs. It was found that, just over 35% have taken courses related to disablist bullying previously. Thus, only a little more than half of the participants know websites, associations, or professionals that could help them in situations of disablist bullying. Finally, 63.5% consider that the way of reporting bullying situations at school or at work is insufficient.

**TABLE 7:** Results on direct interaction/resources/organization.

	**Yes**	**No**
I am aware of the steps that can be taken when bullying occurs	62.5%	37.5%
I have attended courses related to the prevention of disablist bullying	36.5%	63.5%
I have had help from someone other than a member of staff (e.g., Doctor, Psychologist, Counselor, etc.)	65.6%	34.4%
I believe that the way in which the school/workplace reports bullying is insufficient	63.5%	36.5%
I know web pages, associations, and professionals that can help me in situations of disablist bullying	52.1%	47.9%

In the last factor on levels of interaction, resources, and organization, the average was 7.2 (*SD* = 1.32). The average for women was 7.15 (*SD* = 1.33), while for men, it was 7.25 (*SD* = 1.31). This difference was not significant, *t*(94) = −0.35, *p* = 0.724, *d* = 0.08. However, significant differences were found in terms of age, *F*(1,3) = 3.42, *p* = 0.02, μ^2^ = 0.06. As shown in the following Table 8, after performing the Bonferroni test (equal variances are assumed with Levene’s test), there were differences between the 21–22 years (*M* = 6.25, *SD* = 1.36) age group and the 15–17 years age group (*M* = 8, *SD* = 1.41).

**TABLE 8:** Differences according to age in the dimension “levels of direct interaction/resources/organization”.

**(I) Age**	**(J) Age**	**Mean difference (I-J)**	**Standard error**	**Sig.**
15–17	18–20	0.60	0.56	1.0
	21–22	1.75	0.58	0.02
	+23	0.77	0.48	0.66
18–20	15–17	−0.60	0.56	1.0
	21–22	1.15	0.49	0.13
	+23	0.17	0.37	1.0
21–22	15–17	−1.75	0.58	0.2
	18–20	−1.15	0.49	0.13
	+23	−0.98	0.40	0.10
+23	15–17	−0.77	0.48	0.66
	18–20	−0.17	0.37	1.0
	21–22	0.98	0.40	0.1

### Results After Training

The following Table 9 highlights the main changes obtained after the completion of the training.

**TABLE 9:** Differences between before and after training.

**Attitudes, personal opinion**		

**Items**	**Pre-test**	**Pos-test**
People with SEN/D are more at risk of bullying than other people	77.1%	87.2%
I think that bullying is a normal thing that happens to people	42.7%	20.5%
**Personal experience**		
I have done courses about bullying	34.4%	73.1%
**Skills to prevent and cope**		
If a classmate or friend makes an annoying comment to me or makes me feel upset, I’ll tell them	83.3%	89.7%
I know how to say no when a classmate or someone asks me to do something and I don’t want to do it	75%	78.2%
**Knowledge about bullying**		
I know the differences between having a conflict with someone and being bullied	62.5%	74.4%
Bullies may have behavior problems and low self-esteem	82.3%	84.6%
Bullying is a power imbalance, the bully wants to be in charge	84.4%	92.3%
Bullying is when someone wants to hurt you on purpose by doing or saying something	83.3%	89.7%
Bullying is when your boss, teacher, good friend, support staff, or parents tell you off because you have done something that you should not have done	36.1%	26.9%
**Levels of direct interaction/resources/organization**		
I am aware of the steps that can be taken when bullying occurs	62.5%	85.9%
I have attended courses related to the prevention of disablist bullying	36.5%	64.1%

As shown in the table above, many aspects have improved after the training received by the students. Above all, block 3 on prevention and management skills and block 4 on knowledge of bullying stand out. These two blocks were an essential part of the work with the students. It is noteworthy that 42.7% of the participants thought that being bullied is normal and usual in the beginning, but after the training only 20.5% thought so.

## Discussion and Conclusion

Bullying is a reality that we cannot ignore; a problem that has increased due to the use of technology. The data speaks of a situation that occurs more than we would like. Moreover, this problem is aggravated when we mention people with SEN/D. Since 1989 it has been observed that these people are the most vulnerable to this type of action (O’Moore and Hillery, 1989). It is a reality that we continue to observe today (González Contreras et al., 2020).

This situation is the starting point of the DisAbuse project, a project financed with European funds through the Erasmus+ program that was developed in four institutions in four different countries: Ireland, Spain, Portugal, and Italy. The aim of this project was the development of a program to help reduce harassment of people with SEN/D. This was based on previous experiences developed by specific section of people in the participating countries and the analysis of the situation based on a questionnaire. The data obtained from this analysis are those presented here.

People with SEN/D have participated in this study, the majority of whom were people with intellectual disabilities. Therefore, the questionnaire, after a process of expert judgment and group dynamics, was adapted to the special needs of the participants. Therefore, the questions presented here were answered by the subjects by “yes” and “no” and, on some occasions, were helped by the researchers, who always respected their privacy and allowed them to answer according to their own experience.

First, they were asked about certain attitudes toward bullying and their views on these situations. Most participants (77% in the pre-test, 87% in the post-test) indicated that people with SEN/D are more at risk of bullying than other people. This self-perception of risk is consistent with data obtained in previous research about the real statistics of disablist bullying (O’Moore and Hillery, 1989; Bourke and Burgman, 2010; Blake et al., 2012; Christensen et al., 2012; Naylor et al., 2012; Bear et al., 2015; González-Contreras, 2017; Menesini and Salmivali, 2017; Pinquart, 2017; Schrooten et al., 2017; Jenaro et al., 2018; Maguire et al., 2019).

It is remarkable that almost half of them consider bullying to be normal, and a large proportion of them believe that the bully does not care about the victim’s feelings. These data relate to the fact that nearly three-quarters of the participants believe that people with SEN/D are more at risk of being bullied. This last data shows that people with SEN/D are aware of being more vulnerable to bullying as shown by other researches (González Contreras et al., 2020; Suárez-García et al., 2020). They also estimated that Internet use may increase the likelihood of being harassed.

As for their personal experience related to bullying, a large percentage knows people who have been bullied. In contrast, only a third have taken courses related to bullying. What is more worrying is that almost half of the participants consider that it is not their problem when someone else is left out of the group, so they do not consider that to be a type of bullying.

As for the skills to prevent bullying, they may suffer themselves, as in general they generally tend to avoid these situations. Thus, they consider that the rules on bullying is important, as they are able to enlighten people on situations of bullying. It was found that they respect everyone equally and are able to resolve conflicts with friends without fighting. However, when it came to aspects more related to them, the percentages went down. Thus, a quarter of the participants did not know how to say “no” when asked to do something they do not want to do. Likewise, a not inconsiderable percentage were unable to speak to a friend when a comment bothered them. It is a complicated situation since the person feels intimidated and is not able to talk about it.

One of the essential aspects of the project was to improve the participants’ knowledge of this issue; so they were asked about some essential elements. Firstly, it was noted that many participants did not know the difference between bullying and conflict. Secondly, it is highlighted that most of them seem to know the main characteristics of bullying: power imbalance, intentionality, and repetition. However, this idea contrasts with the fact that a little more than a third of the participants consider that when they are reprimanded, they are also being bullied.

Another aspect considered important within the project is that people with SEN know the path they should follow if they suffer from bullying or that they know the resources available to them in these situations. Before the completion of the courses within the project, just over a third did not know what steps to take in case of these problematic situations. Moreover, only half of them knew websites, associations, or professionals that could help them in case of disablist bullying. Certainly, these two aspects are preceded because the vast majority has not attended courses or have been trained for the prevention of bullying.

In relation to sex, only significant differences were found in the “Skills to prevent and cope” factor. In this case it is the males who showed better coping and prevention skills. No studies have been found that show differences in this population. However, in the paper presented by Coppari et al. (2019) it is women who show more coping skills. With regard to age, only significant differences were found in the latter factor, with this difference being mainly between the 21–22 and 15–17 age groups. One of the most important data of the work is reflected in the improvement of some data obtained before and after carrying out the training program. These data help to understand that this type of training is necessary for those students who have SEN, whether they have a disability or not. No studies of training programs to prevent bullying in people with SEN/D have been found. As stated in the work of Gómez and Navarro (2017), it is necessary to extend the training, not only to this group of people, but also to the rest, in order to learn to respect the differences between people.

In conclusion, the finding presented here shows a reality. Bullying of young adults with SEN/D occurs more frequently than it should, and they do not have sufficient skills, knowledge, and resources to deal with these situations. Our data are on the similar lines with González-Contreras (2017); Pinquart (2017), and Jenaro et al. (2018) as people with SEN have a self-perception that such people suffers more bullying than the rest of the population. It is necessary to continue implementing training and prevention programs from a young age for this population group. Our results also reflect that of Maguire et al. (2019), demonstrating the value of the educational intervention with teachers, professionals, and students with SEN. We agree with Maguire et al. (2019) and with Menesini and Salmivali (2017) who remark on the relevance of training as useful and effective strategy to prevent bullying. These authors mention the relevance of working with all the groups involved in the prevention of bullying, and not only educational intervention with people with SEN or with the students in general. Teachers, parents, and professionals must be also considered as key actors in these processes and they must have a main role to avoid disablist bullying. The experience of Disabuse is also in line with other funded projects we have cited above, which focus on the interest on fostering good practices and improving the inclusion of people with SEN.

Occasionally, people with SEN/D are not aware of the situation they are living in; therefore, parents and professionals who are with them must also have training and resources to confront and solve this problem. Thus, it is necessary to improve the inclusion of people with SEN in order to better understand people with these needs and reduce the bullying that they face (Cook et al., 2020).

The main limitation of Disabuse project was the time, because we worked for 3 months with the participants in all the countries. According to Menesini and Salmivali (2017), the intensity and duration of the training programs is one of the most relevant factors to assure its impact. We are aware that it is always necessary to continue working with the educational agents and to assess the results after a substantial amount of time, to assure its effectiveness. Furthermore, it would be relevant to work with parents (Bourke and Burgman, 2010; Ttofi and Farrington, 2011), however, it is usually exceedingly difficult to involve them in long-lasting training programs.

## Data Availability Statement

The raw data supporting the conclusions of this article will be made available by the authors, without undue reservation.

## Ethics Statement

The studies involving human participants were reviewed and approved by the Comisión Ética de Investigación de la Universidad de Murcia. Written informed consent to participate in this study was provided by the participants’ legal guardian/next of kin.

## Author Contributions

All authors have contributed equally to each and every part of this manuscript, involved in the bibliographic search and review, as well as in the methodological design and statistical data analysis, in charge of data collection, and participated in the interpretation and drafting of the results, as well as in the discussion and conclusion of the study carried out.

## Conflict of Interest

The authors declare that the research was conducted in the absence of any commercial or financial relationships that could be construed as a potential conflict of interest.

## Footnotes

1 www.time-to-change.org.uk

2 www.srtrc.org

3 www.seemescotland.org

4 https://aepae.es

5 http://www.antibullying.eu

6 http://iamnotscared.pixel-online.org

7 http://www.sonetbull.eu

8 https://eubully.eu

9 http://www.includ-ed.eu/es

10 www.disabuse.eu
